# Tachycardia-Induced Cardiomyopathy Following Prolonged Ritodrine Infusion During Pregnancy: A Case Report

**DOI:** 10.7759/cureus.76465

**Published:** 2024-12-27

**Authors:** Masahiro Nakao, Miho Izawa, Itaru Takamisawa, Chinami Horiuchi, Azumi Ohmori, Shinji Katsuragi

**Affiliations:** 1 Department of Obstetrics and Gynecology, Sakakibara Heart Institute, Fuchu, JPN; 2 Department of Obstetrics and Gynecology, Mie University Graduate School of Medicine, Tsu, JPN; 3 Department of Cardiology, Sakakibara Heart Institute, Fuchu, JPN; 4 Department of Obstetrics and Gynecology, University of Miyazaki, Miyazaki, JPN

**Keywords:** beta-adrenergic agonists, cardiomyopathy, pregnancy, ritodrine, tachycardia

## Abstract

Preterm birth remains a leading cause of neurodevelopmental disability in offspring, prompting various preventive measures. However, controversies persist surrounding these approaches, particularly regarding beta-mimetic drugs. In Japan, it remains a concerning reality that ritodrine infusion continues to be used for long-term tocolysis in preterm labor, despite the warning issued by the US Food and Drug Administration. Growing evidence suggests that the use of beta-adrenergic agonists during pregnancy is associated with an increased risk of maternal cardiac systolic dysfunction. Furthermore, tachycardia-induced cardiomyopathy (T-CM), a rare etiology of cardiomyopathy, can also be triggered by beta-adrenergic stimulation. Here, we present a case of a 35-year-old pregnant woman without prior arrhythmias who developed T-CM following prolonged ritodrine infusion for preterm labor. The patient experienced persistent, exacerbated tachycardia under continuous ritodrine infusion starting at 20 weeks of gestation and developed supraventricular tachycardia with an impaired left ventricular ejection fraction of 30% and an elevated N-terminal prohormone of brain natriuretic peptide (NT-proBNP) level of 2,122 pg/mL, at the time of transfer at 36 weeks of gestation. Administration of adenosine and verapamil revealed atrial tachycardia or flutter, and the patient was diagnosed with tachycardia-induced cardiomyopathy, which was confirmed by a dramatic improvement in systolic function following maternal heart rate control using a bisoprolol patch. The patient delivered a healthy female via cesarean section at 37 weeks of gestation and was discharged from the hospital with favorable maternal and neonatal outcomes. This report highlights another potential risk of maternal cardiac deterioration due to beta-adrenergic effects, emphasizing the importance of reconsidering the long-term use of ritodrine infusion, as well as the need for dose titration, discontinuation, or switching to the alternative agent in cases of persistent or exacerbated tachycardia. Careful monitoring, including more frequent daily checkups to assess pulse rate and respiratory changes, along with the use of early warning systems and occasional BNP or NT-proBNP measurements, should be considered to ensure the timely detection of deterioration and the implementation of earlier preventive interventions.

## Introduction

Preterm birth remains a leading cause of neurodevelopmental disability in offspring [1]. Despite various options for preventative measures, there still remains debate surrounding all these approaches. Beta-mimetic drugs, such as ritodrine and terbutaline, have been used as tocolytic agents for preterm labor, acting as uterine muscle relaxants through beta-2 receptor stimulation [2]. The restriction on the use of beta-mimetics for long-term tocolysis (i.e., beyond 48 hours) became more pronounced following a warning issued by the US Food and Drug Administration, which highlighted serious risks such as maternal tachycardia, pulmonary edema, and myocardial ischemia [3]. Although the use of ritodrine for long-term tocolysis has declined in Japan, it remains a concerning reality that many facilities across various regions continue its use. The Japanese obstetric practice guidelines do not explicitly recommend against its long-term use [4], even in the updated version [5]. While acknowledging the lack of evidence supporting its efficacy and the potential for serious adverse events, the guidelines state that when continuing ritodrine infusion beyond 48 hours or switching to oral administration after discontinuing the infusion, careful consideration should be given to the feasibility of dose reduction or cessation before making a clinical decision [5].

Growing evidence suggests that the use of beta-adrenergic agonists during pregnancy is associated with an increased risk of maternal cardiac systolic dysfunction. A retrospective analysis in a Taiwanese cohort showed the significant association between ritodrine infusion and an increased risk of heart failure (adjusted odds ratio, 5.58; 95% confidence interval, 1.47-3.32) [6]. Additionally, a surveillance study on maternal death in Japan revealed that maternal death due to peripartum cardiomyopathy (PPCM) was more prevalent in patients who received ritodrine infusion compared to those who did not (12.5% vs. 0.3%, p<0.01) [7]. However, further investigation is needed to elucidate the mechanisms underlying this condition and to explore other potential etiologies of pregnancy-associated cardiomyopathy.

Tachycardia-induced cardiomyopathy (T-CM) is a rare cause of left ventricular (LV) dysfunction resulting from an elevated heart rate (HR), typically triggered by preexisting tachyarrhythmias, such as atrial fibrillation, incessant supraventricular tachycardia (SVT), or ventricular tachycardia. Clinical manifestations include symptoms of heart failure and palpitations. T-CM is characterized by its reversibility, with LV systolic function normalizing after resolution of the underlying arrhythmia, which confirms the diagnosis [8]. Although beta-mimetic use may be considered a potential trigger for T-CM [6,9], its prevalence remains unclear or may be underestimated [8], and many obstetric care providers are unaware of the potential risks associated with this condition. Here, we present a case of T-CM that developed during pregnancy in a patient with no prior history of arrhythmia, following prolonged ritodrine infusion. Written informed consent was obtained from the patient for the publication of this case report.

## Case presentation

Medical history and pregnancy course

A 35-year-old primiparous Japanese woman, who had no notable medical history or family history of cardiac disease, conceived through in vitro fertilization and received routine prenatal care at her obstetrical clinic. Her most recent annual health screening, including electrocardiograms (ECGs), performed prior to pregnancy, revealed no arrhythmias based on her self-report. At 20 weeks of gestation, she was admitted to the local facility with a diagnosis of threatened miscarriage, presenting with genital bleeding and a 1.5-cm dilated cervix, which prompted the initiation of continuous intravenous (IV) ritodrine infusion for tocolysis. A Shirodkar rescue cerclage was performed at 22+3/7 weeks of gestation, which ultimately required an increase in ritodrine dosage to 200 μg/min. Additionally, she was diagnosed with gestational diabetes mellitus at 24 weeks of gestation and effectively managed with dietary modification along with insulin injection therapy.

Clinical presentation and examination

The patient remained on ritodrine infusion at 200 μg/min until 35 weeks of gestation, during which she reported experiencing palpitations since the initiation of the infusion. A preoperative 12-lead ECG at 21+6/7 weeks of gestation revealed sinus tachycardia at 127 bpm with monomorphic premature ventricular contractions; however, no further cardiac work-up was performed. She was monitored with once-daily vital sign checks and weekly evaluations of blood cell counts and biochemical parameters. Her pulse rate remained persistently tachycardic in the 120s at rest and occasionally escalated to the 140s-150s since 34 weeks of gestation, without a cardiac workup during this period. She eventually reported exacerbated palpitations on the day before the transfer at 35+6/7 weeks of gestation and was confirmed to have a pulse rate of 180-200 bpm. IV ritodrine was discontinued, and the patient continued to be monitored. However, the palpitations persisted overnight. Subsequently, the patient was evaluated by the on-call cardiologist the following morning and was identified with SVT on ECG, cardiomegaly on chest radiograph, and LV systolic dysfunction, with a left ventricular ejection fraction (LVEF) of 30% (reference range, 52%-74%), as assessed by bedside transthoracic echocardiography (TTE). Consequently, she was transferred to our facility, a tertiary center for cardiovascular diseases, for a comprehensive maternal and fetal health assessment.

**Figure 1:**
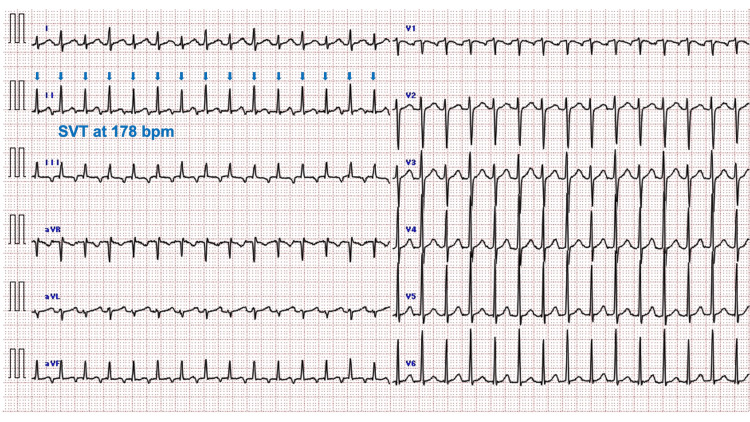
Electrocardiogram at presentation The ECG at presentation shows narrow (<0.12 s), regular QRS tachycardia at 178 bpm, with an absent P wave (indicated by filled arrows). These findings are indicative of SVT, as the narrow, regular QRS complex suggests a supraventricular origin with a reentrant mechanism. Additionally, the absence of P waves indicates retrograde atrial depolarization occurring concurrently with ventricular depolarization. ECG, electrocardiogram; SVT, supraventricular tachycardia

Upon arrival, her vital signs were recorded as follows: body temperature, 36.2℃; HR, 179 bpm (persistent); blood pressure, 104/62 mmHg; respiratory rate, 24 breaths per minute; and SpO_2_, 94% on ambient air. She reported dyspnea at rest, which was classified as New York Heart Association (NYHA) functional class IV based on her self-report. However, orthopnea, jugular venous distention, and pitting edema were not evident on her physical examination. The fetus exhibited a vertex presentation, demonstrating appropriate growth with an estimated fetal weight of 2,810 g, and showed a reactive pattern on electronic fetal monitoring. The ECG confirmed the presence of SVT, characterized by a narrow (<0.12 s), regular QRS tachycardia at 178 bpm, suggesting a supraventricular origin with a reentrant mechanism, and the absence of P waves indicating retrograde atrial depolarization occurring concurrently with ventricular depolarization (Figure 1).

**Figure 2:**
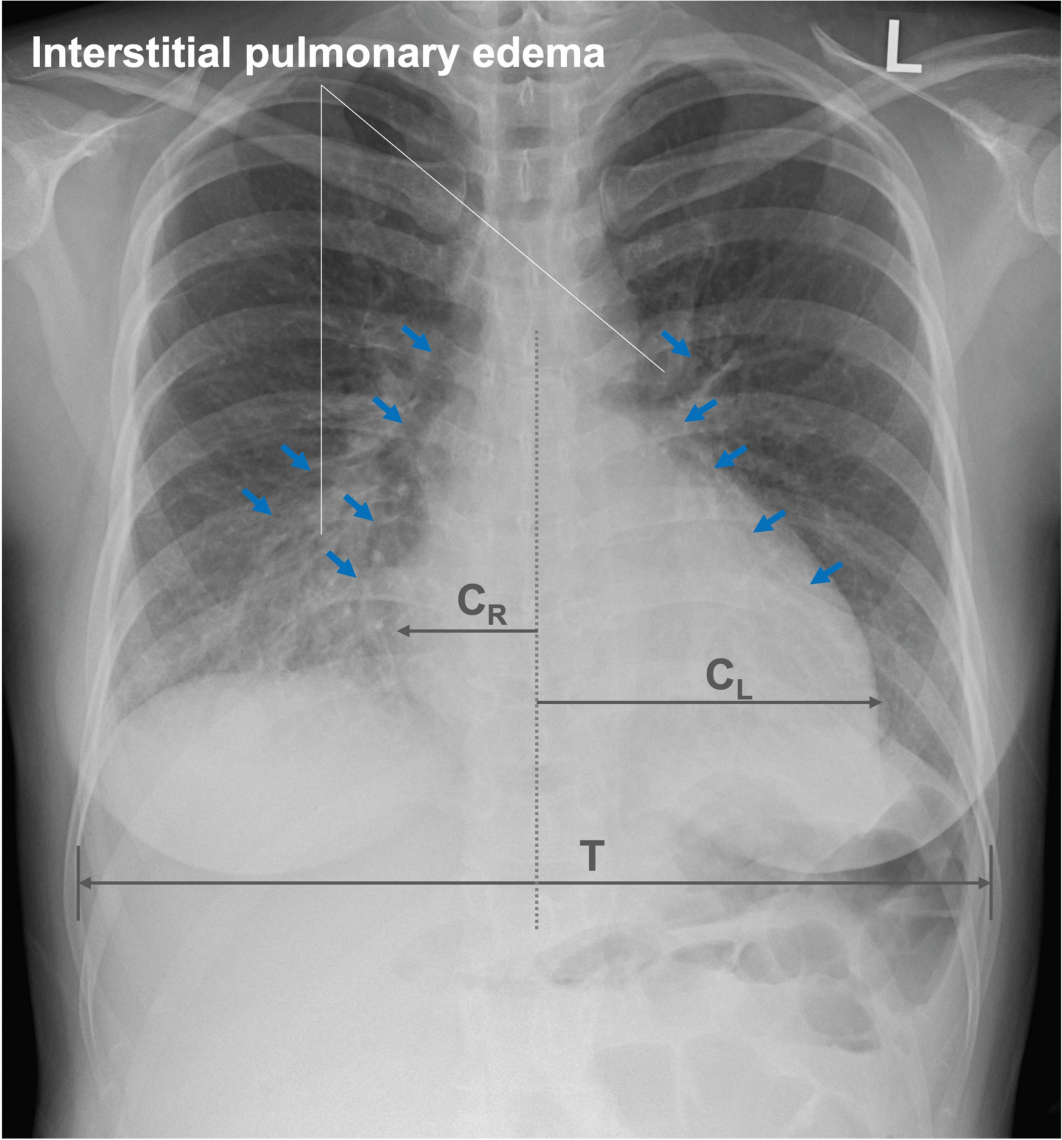
Chest radiograph at presentation The posteroanterior chest radiograph at presentation demonstrates cardiomegaly, with a CTR of 53% (calculated using the formula (C_R_ + C_L_)/T × 100), and bilateral interstitial pulmonary edema, characterized by air bronchograms (indicated by filled arrows). These findings suggest pulmonary congestion due to heart failure (T-CM). CTR, cardiothoracic ratio; C_L_, horizontal left cardiac diameter; C_R_, horizontal right cardiac diameter; T, maximal horizontal thoracic diameter; T-CM, tachycardia-induced cardiomyopathy

Furthermore, the chest radiograph indicated cardiomegaly, with a cardiothoracic ratio (CTR) of 53% (reference range, 35%-50%), and bilateral interstitial pulmonary edema, characterized by the presence of air bronchograms (Figure 2).

**Figure 3:**
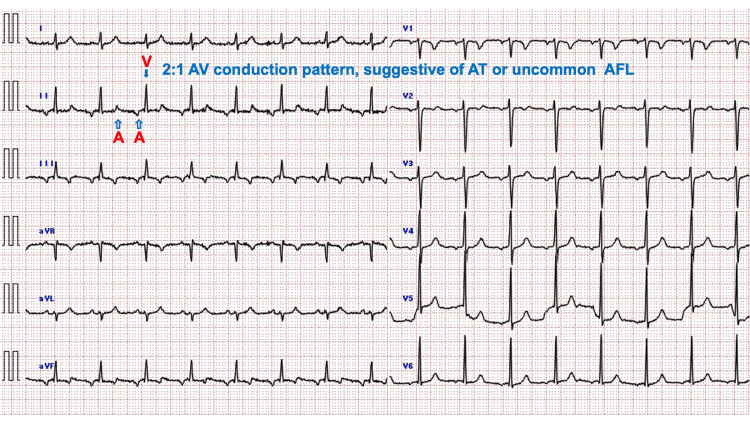
Electrocardiogram after the slow intravenous infusion of verapamil hydrochloride The ECG following IV verapamil infusion illustrates waveforms demonstrating a 2:1 conduction pattern of atrial (denoted by empty arrows labeled “A”) and ventricular (indicated by a filled arrow labeled “V”) waves at a rate of 99 bpm. This pattern suggests the presence of AT or an uncommon form of AFL, both of which are consistent with the etiologies of T-CM. AFL, atrial flutter; AT, atrial tachycardia; AV, atrioventricular; ECG, electrocardiogram; IV, intravenous; T-CM, tachycardia-induced cardiomyopathy

TTE revealed diffuse LVEF impairment at 30.3%, accompanied by a fractional shortening of 19.7% (reference range, 28%-42%). There was no evidence of left atrial or ventricular dilation (left atrial diameter, 3.6 cm, reference range, 2.7-4.0 cm; end-diastolic/systolic LV diameter, 4.8/3.8 cm, reference range, 2.0-3.7/3.5-5.3 cm), LV hypertrophy (septal and posterior wall thickness, 0.8 cm and 0.75 cm, reference range, 0.6-1.0 cm for both), nor any valve regurgitation. The paroxysmal SVT ceased after the administration of a 10-mg IV bolus of adenosine triphosphate, followed by a 5-mg slow IV infusion of verapamil hydrochloride, resulting in a 2:1 atrioventricular conduction. This pattern was suggestive of either atrial tachycardia (AT) or an uncommon form of atrial flutter (AFL) (Figure 3).

Laboratory data at presentation revealed an elevated N-terminal prohormone of brain natriuretic peptide (NT-proBNP) level of 2,122 pg/mL (reference, ≤125 pg/mL), but showed no evidence suggestive of underlying etiology for heart failure, such as ischemic or inflammatory changes, hypokalemia, or hyperthyroidism. Laboratory findings included normal levels of white blood cell count (8,900/μL; reference range, 3,500-9,100/μL), creatine kinase (49 U/L; reference range, 45-163 U/L), potassium (4.0 mEq/L; reference range, 3.6-5.0 mEq/L), thyroid-stimulating hormone (1.13 μIU/mL; reference range, 0.50-5.00 μIU/mL), free triiodothyronine (2.30 pg/mL; reference range, 2.30-4.30 pg/mL), and free thyroxine (0.98 ng/dL; reference range, 0.90-1.70 ng/dL).

Treatment and clinical course

**Figure 4:**
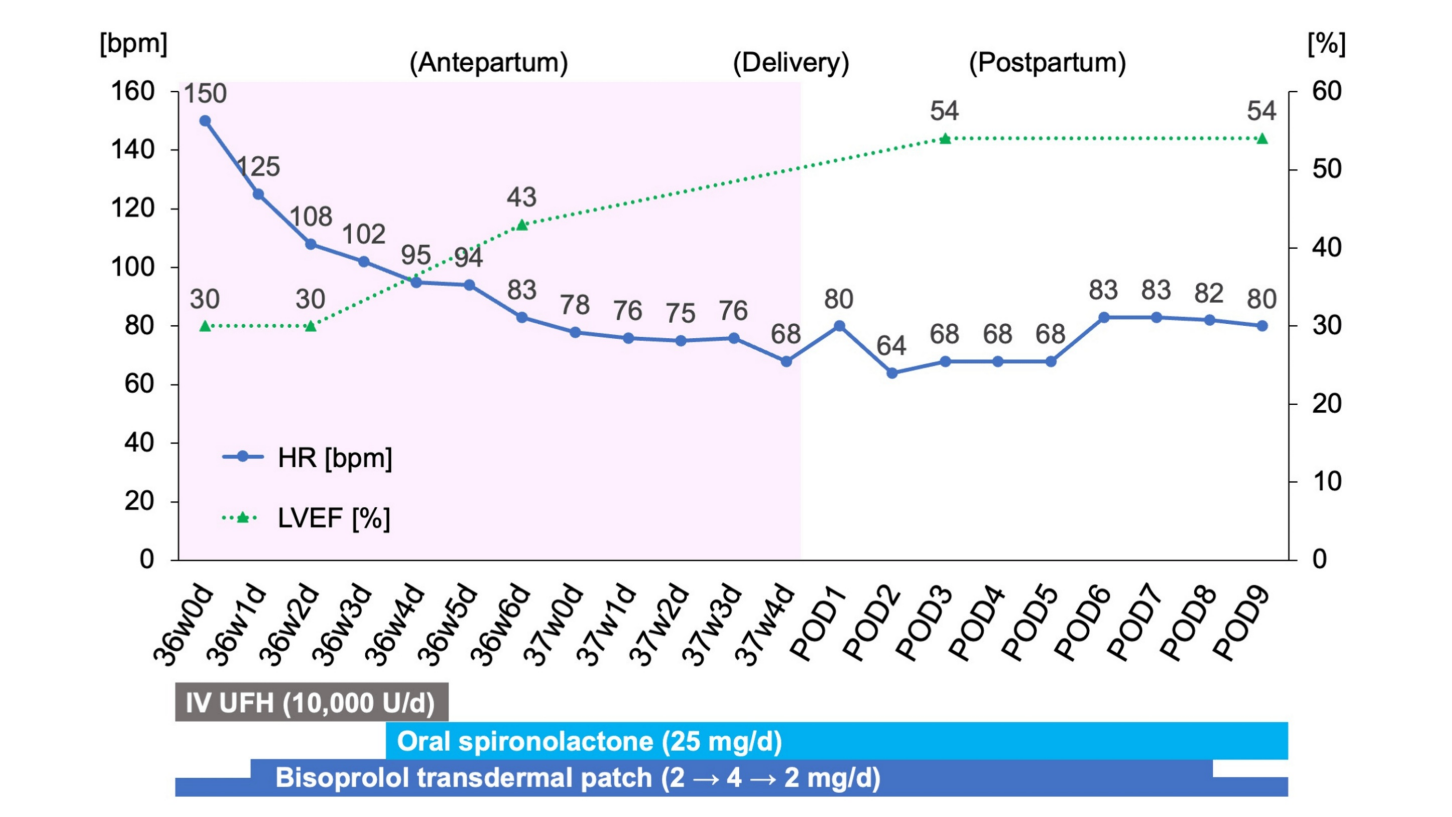
An overview of the clinical course during hospitalization The image shows the recovery of LVEF values (illustrated by the dotted line) and maternal HR control (illustrated by the solid line) after treatment intervention. HR, heart rate; IV, intravenous; LVEF, left ventricular ejection fraction; POD, postoperative day; UFH, unfractionated heparin

The patient was suspected of T-CM or PPCM, and the initial treatment strategy included maternal HR control using a 2-mg bisoprolol transdermal patch, which was increased to 4 mg on the following day, supplemented by a 5-mg IV slow infusion of verapamil, administered as needed. As the onset of LV impairment and tachyarrhythmia remained unclear, potentially occurring more than 48 hours prior to referral, prophylactic anticoagulation was initiated with a daily IV infusion of 10,000 units of unfractionated heparin (UFH), due to the presumed risk of thromboembolism. Additionally, an oral dose of 25 mg of spironolactone, a potassium-sparing diuretic, was chosen to mitigate aldosterone-mediated myocardial remodeling. Subsequently, the baseline HR was controlled, decreasing from approximately 180 bpm to below 90 bpm, with maternal heart rhythm returned to sinus rhythm without side effects or further complications. Follow-up testing at 36+6/7 weeks of gestation demonstrated improved systolic function (LVEF, 43%) and a decline in NT-proBNP levels to 255 pg/mL, supporting the diagnosis of T-CM. Induction of labor commenced after the removal of cerclage stitches at 37 weeks of gestation, resulting in a cesarean delivery due to induction failure. The newborn, a healthy female weighing 3,154 g, exhibited Apgar scores of 8 at one minute and 9 at five minutes, with an umbilical arterial pH of 7.32. Strict fluid management and urine output monitoring, combined with continued use of the bisoprolol patch throughout the postpartum period, effectively maintained the patient’s stable cardiac status, with an LVEF of 54%, and no recurrence of tachycardias or heart failure, as confirmed by continuous ECG monitoring and TTE. Both the mother and the offspring recovered well, leading to their discharge from the hospital on the ninth postoperative day (Figure 4).

At one month post-delivery, there were no signs of tachycardia or heart failure recurrence, as confirmed by a normal chest X-ray, normal sinus rhythm at 61 bpm on ECG, and preserved LVEF of 54% on TTE. NT-proBNP levels had decreased to 131 pg/mL. The patient remained in NYHA functional class I, and outpatient monitoring was continued thereafter, with plans to discontinue bisoprolol and schedule a follow-up Holter ECG at six months postpartum to assess the need for catheter ablation.

## Discussion

In this report, we have described a rare case of T-CM in a pregnant woman with de novo arrhythmias, likely associated with prolonged ritodrine infusion. T-CM is diagnosed based on the presence of tachyarrhythmia, partial or complete recovery of LV function upon restoration of sinus rhythm or rate control, and the exclusion of other causes of heart failure [8]. Determining whether tachyarrhythmia is the primary cause of heart failure or a secondary result of cardiomyopathy from another origin (e.g., PPCM, which is also diagnosed by excluding other causes of heart failure) is challenging. However, the timeline of heart failure in the present case, characterized by an impaired LVEF of 30% following persistent and exacerbated tachycardia that normalized dramatically with maternal HR control from 180-200 bpm at presentation to within the normal range, aligns more closely with the features of T-CM than PPCM [8], which is not directly linked to such arrhythmias. Furthermore, the known effects of beta-adrenergic stimulation by ritodrine during pregnancy, including maternal tachycardia, along with ST-segment depression, T-wave flattening or inversion, and prolongation of the QT-interval [9], could explain its association with an increased risk of maternal arrhythmia [6] and may support a causal relationship in this case.

Notably, the patient developed T-CM despite the absence of known baseline cardiovascular risks, aligning with class 1 of the modified World Health Organization classification [10]. The previous literature on T-CM during pregnancy has highlighted the presence of an arrhythmic background [11]. However, there is uncertainty regarding the actual baseline features in the absence of the 12-lead or Holter ECG data prior to conception, as this is based solely on the patient’s self-report. Nonetheless, it is acknowledged that both supraventricular and ventricular arrhythmia may intensify during pregnancy, especially among women of advanced maternal age [12,13]. This escalation is theoretically attributed to elevated plasma catecholamine levels, increased adrenergic receptor sensitivity, atrial stretch, and end-diastolic volume [14]. Furthermore, symptomatic heart failure can develop within a few weeks, even in the case of mild tachycardia [15]. Therefore, it is conceivable that a latent arrhythmia was activated via beta-adrenergic effects, leading to impaired LV contractility in the present case.

The prognosis for T-CM is generally favorable when effective control of the underlying arrhythmia is achieved. However, safe options for anti-arrhythmic agents during pregnancy are limited (e.g., amiodarone should be avoided due to the risk of fetal thyroid insufficiency). Although electrical cardioversion is a viable option when the Valsalva maneuver or pharmacological approaches fail, or in the case of hemodynamic instability, it should be preceded by anticoagulation, in this instance, to minimize the risk of stroke [16]. Additionally, catheter ablation during pregnancy is often avoided due to concerns about ionizing radiation exposure and technical challenges [10]. It is important to note that histopathological and immunological alterations in T-CM patients, including cardiomyocyte apoptosis, suggest an irreversible aspect [17]. This may play a role as an underlying mechanism for the rare occurrence of sudden cardiac death [15,18]. Before establishing its clinical implications, further studies on the long-term pathophysiological effects of beta-agonist use on cardiac function are crucial to elucidate the underlying mechanism and determine the long-term predictive value, as well as the dosages and durations that pose a risk for myocardial dysfunction.

Nevertheless, careful monitoring of maternal tachycardia is essential, including a thorough evaluation of the associated risks, such as cardiopulmonary deterioration observed in the present case, alongside the potential benefits from its long-term use. The fact that the present case did not receive a cardiac workup before 35 weeks of gestation warrants further exploration. Palpitations with mild tachycardia are commonly observed after initiating ritodrine infusion, which can make it challenging for obstetric care providers to promptly recognize the need for a cardiac workup. However, her HR repeatedly exceeded 140 bpm at 34 weeks of gestation, a level associated with an increased risk of life-threatening events within eight hours [19]. More frequent daily checkups to assess pulse rate and respiratory changes, along with the use of a warning scoring system, may help identify warning tachycardia earlier [20], enabling prompt cardiac workup and potentially mitigating the risk of severe outcomes. Occasional BNP or NT-proBNP measurements may also be useful, given the absence of typical physical signs of heart failure in this case, such as orthopnea, jugular venous distention, and pitting edema [21]. Additionally, dose reduction or discontinuation of ritodrine or switching to an alternative option (e.g., magnesium sulfate infusion), should at least be considered in cases of persistent or exacerbated tachycardia, as stated in the practice guidelines [4,5].

## Conclusions

In summary, we presented a case of T-CM in a pregnant woman with no prior history of arrhythmias, developing after prolonged ritodrine administration. Key diagnostic factors included severe LV dysfunction in the context of tachyarrhythmia, with marked recovery following HR control. Although baseline information is limited, this report highlights the risk of cardiac deterioration due to the beta-adrenergic effects of beta-agonists (e.g., ritodrine, terbutaline), emphasizing the need to reconsider their prolonged use for preterm labor and to consider dose reduction or discontinuation in cases of persistent or exacerbated tachycardia. Careful monitoring, including more frequent checkups for pulse rate and respiratory changes, use of early warning systems, and occasional BNP or NT-proBNP measurements, can help detect tachyarrhythmia or deterioration early and enable preventive actions. Further research is needed to understand the long-term effects of beta-adrenergic agents on cardiac function and to identify the dosages and durations that risk myocardial dysfunction.
